# Suppression of Peroxiredoxin 4 in Glioblastoma Cells Increases Apoptosis and Reduces Tumor Growth

**DOI:** 10.1371/journal.pone.0042818

**Published:** 2012-08-15

**Authors:** Tae Hyong Kim, Jieun Song, Sheila R. Alcantara Llaguno, Eric Murnan, Sandya Liyanarachchi, Kamalakannan Palanichamy, Ji-Yeun Yi, Mariano Sebastian Viapiano, Ichiro Nakano, Sung Ok Yoon, Hong Wu, Luis F. Parada, Chang-Hyuk Kwon

**Affiliations:** 1 Dardinger Center for Neuro-Oncology and Neurosciences, Department of Neurological Surgery, The Ohio State University Wexner Medical Center, Columbus, Ohio, United States of America; 2 Solid Tumor Program, The James Comprehensive Cancer Center, the Ohio State University Wexner Medical Center, Columbus, Ohio, United States of America; 3 Department of Developmental Biology, The University of Texas Southwestern Medical Center, Dallas, Texas, United States of America; 4 Human Cancer Genetics Program and Biomedical Informatics Shared Resources, The James Comprehensive Cancer Center, The Ohio State University Wexner Medical Center, Columbus, Ohio, United States of America; 5 Department of Radiation Oncology, The Ohio State University Wexner Medical Center, Columbus, Ohio, United States of America; 6 Department of Molecular and Cellular Biochemistry and the James Comprehensive Cancer Center, The Ohio State University Wexner Medical Center, Columbus, Ohio, United States of America; 7 Department of Molecular and Medical Pharmacology and Institute for Molecular Medicine, School of Medicine, University of California Los Angeles, Los Angeles, California, United States of America; The University of Chicago, United States of America

## Abstract

Glioblastoma multiforme (GBM), the most common and aggressive primary brain malignancy, is incurable despite the best combination of current cancer therapies. For the development of more effective therapies, discovery of novel candidate tumor drivers is urgently needed. Here, we report that peroxiredoxin 4 (PRDX4) is a putative tumor driver. PRDX4 levels were highly increased in a majority of human GBMs as well as in a mouse model of GBM. Reducing PRDX4 expression significantly decreased GBM cell growth and radiation resistance *in vitro* with increased levels of ROS, DNA damage, and apoptosis. In a syngenic orthotopic transplantation model, Prdx4 knockdown limited GBM infiltration and significantly prolonged mouse survival. These data suggest that PRDX4 can be a novel target for GBM therapies in the future.

## Introduction

Glioblastoma multiformes (GBMs) are fatal in most cases, mainly due to tumor infiltration into normal brain structure and resistance to current chemo- and radiotherapies [1]. The median survival of GBM patients is 14.6 months, which has only increased by 2.5 months in the last two and half decades [2]. The current need for innovative cancer therapies is, therefore, particularly high.

Recent cancer genome studies, including a report by the Cancer Genomic Atlas network (TCGA), have clearly identified frequent genetic alterations in human GBMs [3], [4]. Several combinations of these human genetic alterations in mice can recapitulate the human tumor phenotype, indicating that they do cause tumors in human [5]–[7]. More recent genomic analyses have proposed that GBMs can be categorized into a few subtypes, such as proneural, neural, classical, and mesenchymal, based on the type of genes that are expressed or lost [8]–[10]. Despite the different genetic alterations categorizing each tumor subtype and several combinations of causal mutations, most human and mouse GBMs share similar malignant properties, such as high levels of proliferation and tumor cell infiltration. In addition, 80–90% of GBMs harbor deregulated signaling in the PI3K, Rb, and p53 pathways [3]. These phenomena suggest that a remarkable similarity exists among GBMs, and common tumor driver(s) may be present downstream of the cancer gene alterations as genetic or epigenetic changes to directly drive tumor phenotypes. In addition, targeting a tumor driver may improve current cancer treatment.

To identify novel GBM drivers, we employed the Mut6 mouse genetic model (*GFAP-cre*; *Nf1* ^loxP/+^; *Trp53* ^−/loxP^; *Pten* ^loxP/+^) [11] that contains mutations on three of the six most frequently altered genes in human GBMs [3], [4]. Mut6 mice develop diffusely infiltrating high-grade spontaneous astrocytomas (World Health Organization grade 3 and 4 gliomas), including GBMs, with complete penetrance once they reach adulthood [11]. In addition, Mut6 tumors exhibit dysregulation of the PI3K, Rb, and p53 pathways as in human GBMs [3]. From a genomic screening using Mut6 GBM-derived neurospheres, we have identified a putative tumor driver, peroxiredoxin 4 (PRDX4). As an antioxidant protein, PRDX4 reduces intracellular level of reactive oxygen species (ROS), specifically peroxides such as hydrogen peroxide, thereby influencing the signaling pathways that are sensitive to a cellular redox state [12]–[14]. In the present study, we found that PRDX4 expression was increased highly in most human GBMs, as in prostate cancers, lung cancers, and osteosarcomas [15]–[18]. We also report that knocking down PRDX4 expression in physiologically relevant models of GBM resulted in a significant decrease in growth and radio-resistance of GBM cells, suggesting that PRDX4 is a putative GBM driver.

## Results

### PRDX4 was upregulated in both human and mouse GBMs

**Figure 1:**
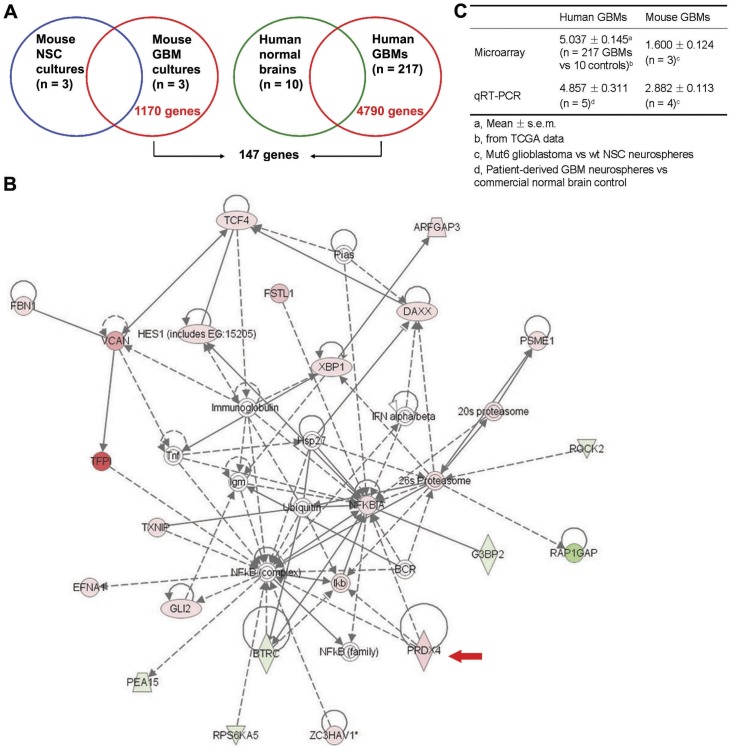
PRDX4 is overexpressed in most GBMs. **A,** Strategy of genomic screening to identify differentially expressed genes in both mouse and human GBMs. **B,** IPA analysis on 147 genes differentially expressed in both mouse and human GBMs identified a network including PRDX4 as top-ranked (score = 42). This network contains 22 genes and is involved in cell death, cellular growth and proliferation, and inflammatory response functions. Various shapes represent genes with different functions. Solid/dash lines represent direct/indirect interactions, respectively. Arrow heads represent activation. Red/green colors represent higher/lower expression, respectively. A red arrow points to PRDX4. **C,** PRDX4 mRNA fold changes in human and mouse GBMs in comparison with normal brain cells. Summary of microarray and qRT-PCR data is shown.

We have previously demonstrated that malignant astrocytomas, including GBM, originate from neural stem cell (NSC) population in *Nf1*; *Trp53*; *Pten* mouse models [19]. We hypothesized that GBM drivers critical for tumor growth or other malignant properties would be differentially expressed in GBM cells compared to their origin cell type. To identify the tumor drivers, we compared gene expression profiles of neurosphere cultures from Mut6 mouse GBMs and normal mouse NSCs using microarray analysis. From the analysis, 1170 genes were differentially expressed only in Mut6 GBMs and not in the normal NSCs (Figure 1A). In parallel with the study, we also analyzed TCGA database (http://tcga-data.nci.nih.gov/tcga/) and obtained 4790 differentially expressed genes in human GBMs compared to normal brain tissues. From the comparison of the 1170 mouse GBM-derived and 4790 human GBM-specific genes, 147 genes were found in both groups (Figure 1A). Ontological analysis using Ingenuity Pathway Analysis (IPA) revealed that the 147 genes were implicated in cellular growth and proliferation, cell death, genetic disorder, cell cycle, neurological disease, and cancer (Figure S1). The top-ranked signaling network among the genes identified by IPA was associated with cell death, cellular growth and proliferation, and inflammatory responses. This network included PRDX4 (Figure 1B and Table S1). Because this network can be relevant in cancer phenotypes and ROS regulation was recently identified as a critical factor in malignant transformation in breast cancers [20], we selected PRDX4 among the 147 genes for further analysis as a putative GBM driver.

Microarray data illustrated that PRDX4 expression was significantly higher in both human (P<1.0×10^−7^) and mouse (Diff score = 40.03, P = 9.93×10^−5^) GBMs than in their respective normal brain counterparts (Figure 1C and Table S1). Notably, PRDX4 expression was more than two-fold increased in most TCGA human GBMs (214/217) than in normal brain tissues (GEO accession number GSE34333). The microarray results were confirmed by qRT-PCR: PRDX4 expression was also significantly increased in both human and mouse GBM neurosphere cultures than in normal brain cells (Figure 1C).

**Figure 2:**
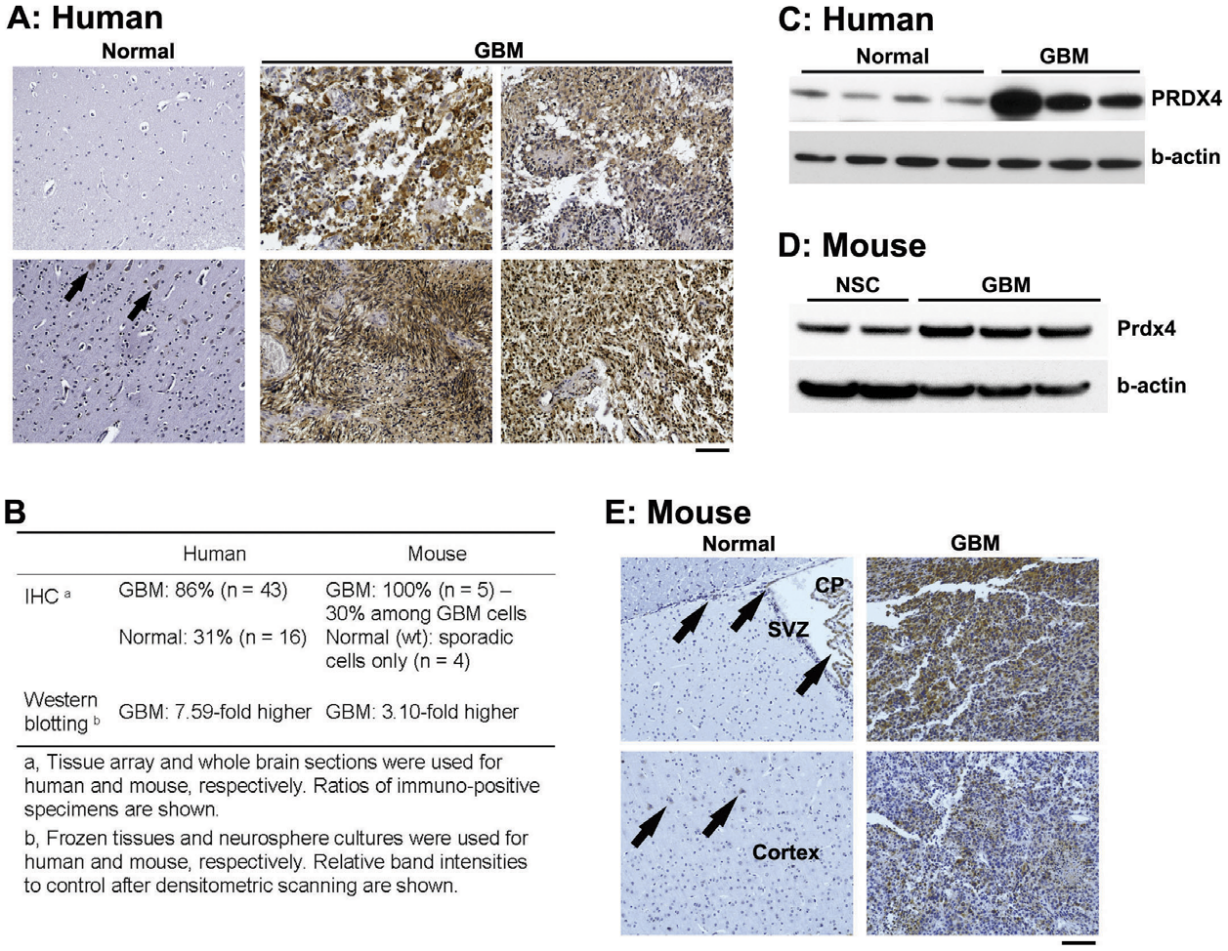
PRDX4 protein is highly expressed in both human and mouse GBMs. **A,** IHC for PRDX4 on human brain sections. Two and four representative images of normal brains (total n = 16) and GBMs (total n = 43), respectively are shown. Arrows point to intermediate intensity of PRDX4 immunoreactivity in normal brains. Scale bar = 100 µm. **B,** PRDX4 protein expression in human and mouse brains. Summary of IHC and Western blotting data is displayed. **C,** Western blotting analysis for PRDX4 on human normal brain versus GBM tissues (frozen specimens). Densitometric scanning reveals 7.59-fold more PRDX4/β-actin ratio in GBM than in normal brain samples. **D,** Representative Western blotting analysis for Prdx4 on wt NSC versus Mut6 GBM neurospheres. Densitometric scanning reveals 3.10-fold more Prdx4/β-actin ratio in GBMs than in NSCs. **E,** IHC for Prdx4 in wt normal versus GBM-containing Mut6 brain sections. Arrows point to Prdx4 immunoreactivity in the SVZ, choroid plexus (CP), and cortex of wt brains. Scale bar = 100 µm. Representative data from n = 5 per group are shown.

Next we examined PRDX4 protein expression in human GBM specimens. Similar to microarray and qRT-PCR data, PRDX4 protein expression was dramatically increased in human GBMs: based on brain tissue arrays, PRDX4 expression was substantially increased in 86% (37/43) of human GBMs, while it was absent in 69% (11/16) of normal control specimen (Figure 2A/B). Western blotting also demonstrated a dramatic upregulation of PRDX4 protein expression in human GBMs compared to that in normal brain tissues (Figure 2B/C). Also in mouse neurosphere cultures, Prdx4 protein levels were >3-fold increased in Mut6 GBMs compared to normal NSCs from the subventricular zone (SVZ) (Figure 2B/D), the major NSC niche [19]. These Western blotting data were confirmed by immunohistochemistry: Prdx4 staining was rarely found in normal mouse brains, except for sporadic cells in the cortex, the SVZ, and the choroid plexus (Figure 2B/E). Within GBM mass from Mut6 mice, approximately 30% of the areas were positive for Prdx4 immunoreactivity (Figure S2 and Figure 2B). These data together demonstrate that PRDX4 overexpression is a common feature associated with GBMs both in human and a mouse model.

### Suppression of PRDX4 expression *in vitro* increased ROS production and the extent of DNA damage and apoptosis while reducing GBM growth

**Figure 3:**
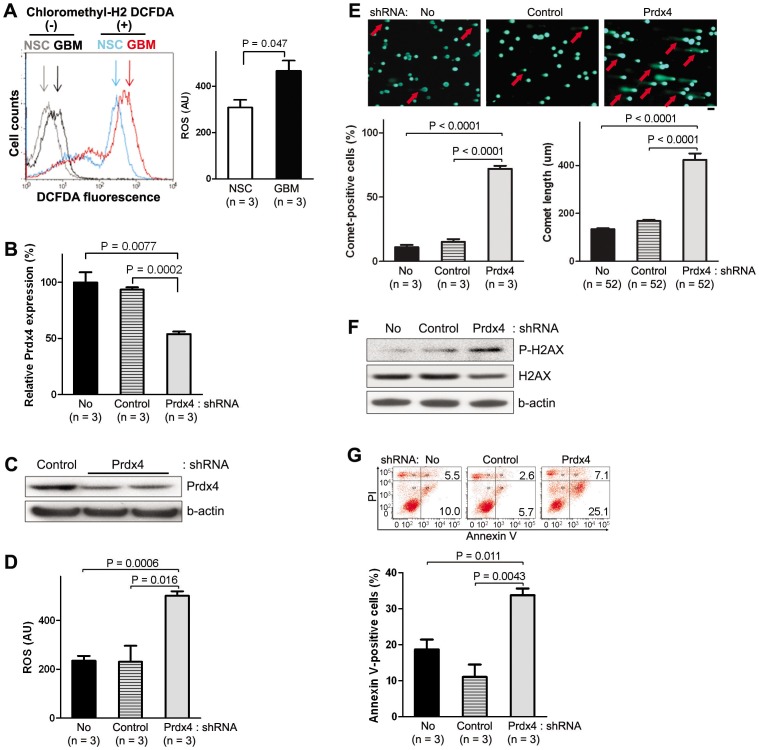
Prdx4 knock-down by shRNA increases ROS, DNA damage, and apoptosis in mouse GBM neurospheres. **A,** Left: FACS analysis after incubation with ROS-sensitive chloromethyl-H_2_ DCFDA represents fluorescent ROS levels in mouse neurosphere cultures three days after seeding. Right: representative assay data from the flow cytometry analysis compare ROS levels in the mouse neurosphere cultures. **B,** Representative qRT-PCR analysis displays relative Prdx4 expression three days after doxycycline treatment to induce shRNA expression in a Mut6 GBM neurosphere culture (ID: Ms6989). **C,** Representative Western blotting analysis for Prdx4 in the mouse GBM neurospheres after shRNA expression. **D,** FACS analysis data after incubation with ROS-sensitive chloromethyl-H_2_ DCFDA compare ROS levels in the mouse GBM neurospheres three days after seeding and doxycycline treatment. **E,** Top: representative GBM neurosphere-forming cells (ID: Ms7080) under comet assay. Arrows point to examples of comet-positive cells. Scale bar = 100 µm. Bottom: comet assay data compare relative comet-positive cells (left) and average comet length (right) in the shRNA groups. **F,** Representative Western blotting analysis for P-H2AX and H2AX in the mouse GBM neurospheres after shRNA expression. Densitometric scanning reveals 3.7- and 1.8-fold more P-H2AX/β-actin ratios in Prdx4 shRNA group than in No shRNA and control shRNA groups, respectively. **G,** Top: representative flow cytometry analysis shows relative numbers of Annexin V-positive mouse GBM cells (ID: 7080) after doxycycline-induced shRNA expression. Upper and lower numbers on each image represent percent of propidium iodide (PI)-positive and –negative cells, respectively. Bottom: summary of three FACS analyses compares relative numbers of Annexin V-positive mouse GBM cells (sum of PI-positive late and PI-negative early apoptotic cells) in the shRNA groups.

It has been reported that tumor suppressor mutations or oncogene activations can increase intracellular ROS levels [21]–[23] and such an increase in ROS can up-regulate expression of antioxidant proteins, including PRDX family members [24], [25]. In support of the reports, we detected a significantly higher ROS level in mouse GBMs than in wt NSC cultures (Figure 3A). Since oxidative stress induced by increased ROS can cause cellular senescence and apoptosis, increased expression of PRDX4 can be a protecting mechanism for GBM cells. We therefore hypothesized that PRDX4 overexpression promotes GBM tumor growth and/or other malignant properties by preventing further ROS accumulation. To test the hypothesis, we knocked down PRDX4 levels in GBM neurospheres using a lentivirus-mediated Tet on/off inducible shRNA expression system that also expresses GFP to mark transduced cells (see Materials and Methods). The efficiency of knockdown was assessed by qRT-PCR. When Prdx4 expression was knocked down by >50% in GBM neurospheres, ROS levels were increased by >2-fold (Figure 3 B–D) in an assay utilizing an ROS-sensitive dye chloromethyl-H_2_ DCFDA that becomes fluorescent when it is oxidized in the cell by ROS [26]. However, when we used an ROS-insensitive probe carboxy-DCFDA, a nonoxidizable analog of chloromethyl-H_2_ DCFDA, as a negative control, there were no significant changes in fluorescence levels (Figure S3), confirming validity of the ROS assay. Taken together, our data suggest that Prdx4 indeed regulates ROS production. Since an increase in ROS can induce oxidative stress, we next asked whether the extent of DNA damage was also increased upon Prdx4 knockdown by performing comet assays. The comet assay is also known as single cell gel electrophoresis assay, and cells that harbor increased amount of DNA breaks display longer comet-like tails, which can be easily detected by fluorescence microscopy after electrophoresis. Indeed, Prdx4 knockdown resulted in 5-fold increase in the number of comet-positive cells and >2-fold increase in the length of the comets (Figure 3E). The increased DNA damage upon Prdx4 knockdown was confirmed by Western blotting for phospho-H2AX (Ser139, P-H2AX), a marker for complex DNA strand breaks [27]: after Prdx4 knockdown, P-H2AX level was increased 1.8–3.7-fold compared to controls (Figure 3F). Also increased was the number of apoptotic cells based on Annexin-V staining: the number of Annexin-V-positive cells increased by 2-fold upon Prdx4 knockdown (Figure 3G).

**Figure 4:**
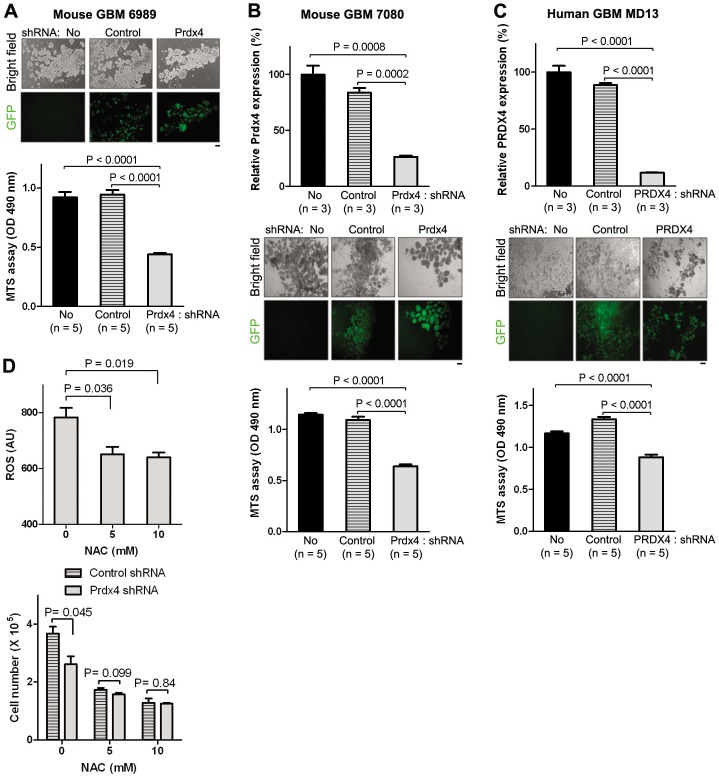
PRDX4 knock-down by shRNA decreases cancer cell growth in both mouse and human GBM neurospheres. **A,** Top: representative mouse GBM neurosphere cells (ID: Ms6989) on assay plates were visualized under bright and fluorescent fields. GFP signals indicate doxycycline-induced shRNA expression. Scale bar = 100 µm. Bottom: representative MTS assay data compare growth of the mouse GBM neurospheres in the shRNA groups three days after doxycycline treatment. **B and C**, Top: representative qRT-PCR data show relative PRDX4 expression after shRNA expression in a mouse (ID: Ms7080, **B**) and a human (ID: HuMD13, **C**) GBM neurosphere cultures. The assays were performed three and six days after doxycycline treatment for mouse and human GBM cultures, respectively. Middle: Representative GBM neurosphere cells on assay plates were visualized under bright and fluorescent fields. GFP signals indicate doxycycline-induced shRNA expression. Scale bar = 100 µm. Bottom: representative MTS assay data show growth of GBM neurosphere cultures three and six days after doxycycline treatment for mouse and human GBM cultures, respectively. **D,** Top: FACS analysis data after incubation with ROS-sensitive chloromethyl-H_2_ DCFDA compare ROS levels in a mouse GBM neurosphere culture (ID: Ms7080) transduced with lentivirus expressing Prdx4 shRNA three days after seeding and treatment with doxycycline and an antioxidant NAC. Bottom: cell counting data from Vi-cell viability analyzer display growth of the GBM neurosphere culture three days after seeding and treatment with doxycycline and an antioxidant NAC.

In line with the increased apoptosis, the rate of cell growth measured by MTS cell viability assay was reduced by 30–50% in multiple GBM neurosphere cultures after PRDX4 knockdown (Figure 4 A–C). A different Prdx4 shRNA sequence also decreased Prdx4 expression and GBM neurosphere growth at similar levels (Figure S4). The knockdown of Prdx4 on the other hand did not increase the cell cycle progression (Figure S5). We next tested whether the GBM cell growth suppression upon Prdx4 knockdown was dependent on increased ROS level by employing an antioxidant N-acetyl cysteine (NAC) that can decrease ROS levels in glioma cells [28]. Treatment with NAC decreased ROS level in spite of Prdx4 knockdown (Figure 4D). In the presence of NAC, GBM cell growth was not significantly changed by Prdx4 knockdown compared to control shRNA group. These results together suggest that PRDX4 is necessary for protecting GBM cells from ROS/DNA damage-induced apoptotic cell death, thereby promoting their overall growth.

### Knocking down PRDX4 expression decreased radiation resistance of GBM cells *in vitro*

**Figure 5:**
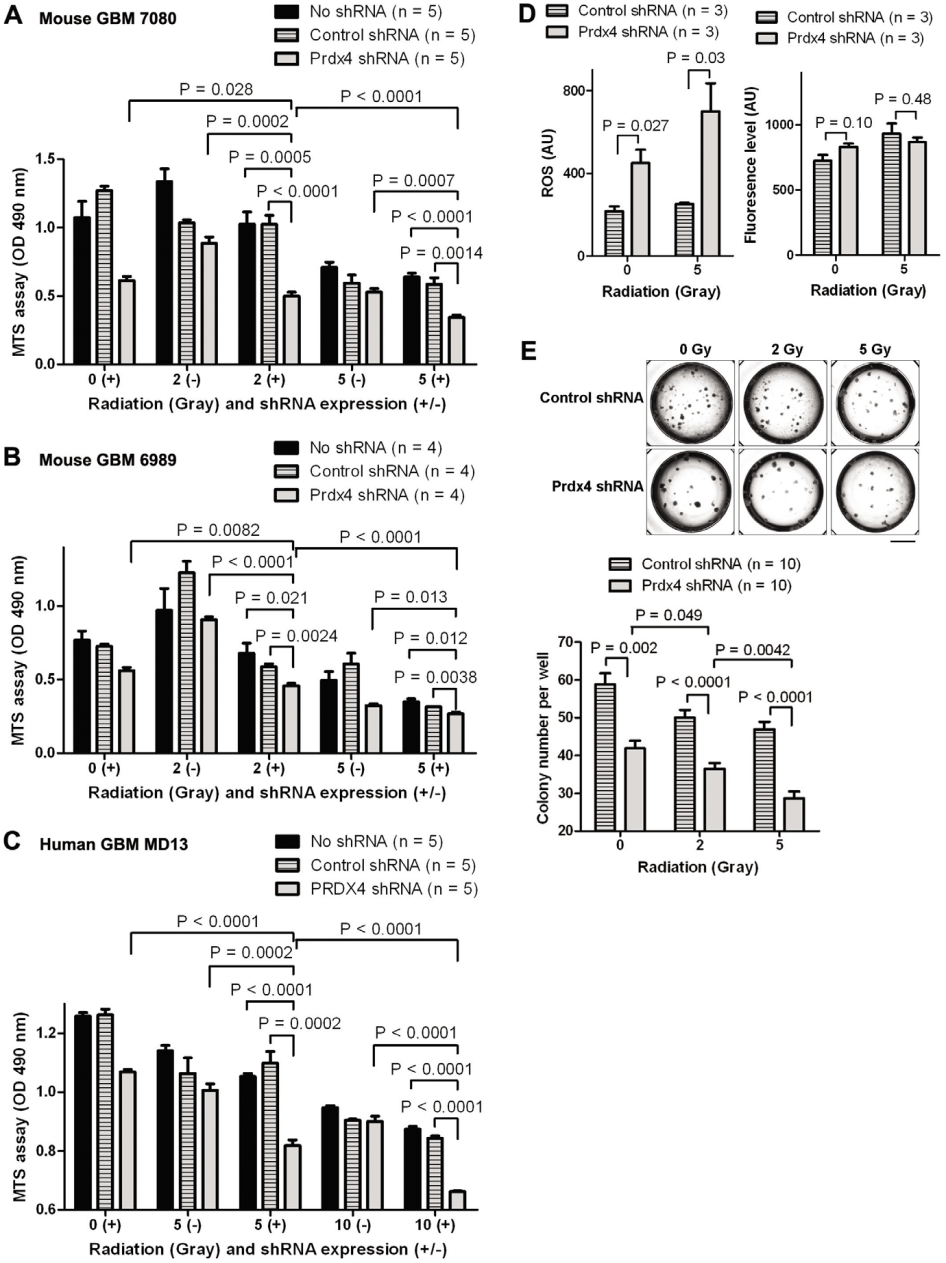
PRDX4 knock-down by shRNA decreases radiation resistance of mouse and human GBM neurospheres *in vitro*. **A–C,** The neurosphere-forming cells were cultured in the presence (+) or absence (−) of doxycycline for three days. After ionizing radiation, the cultures were further incubated for three days and MTS assay was used to assess growth of mouse (**A,** ID: Ms7080; **B,** ID: Ms6989) and a human (**C,** ID: HuMD13) GBM neurosphere cultures. **D,** A mouse GBM neurosphere line (ID: Ms7080) transduced with lentivirus expressing shRNA was cultured in doxycycline-containing media for three days. After ionizing radiation, the culture was further incubated for three days and assessed for ROS levels. Left: FACS analysis data after incubation with ROS-sensitive chloromethyl-H_2_ DCFDA compare ROS levels in the GBM cells. Right: FACS analysis data after incubating the mouse GBM cells with ROS-insensitive carboxy-DCFDA (C-DCFDA) as a negative control of the assay. **E,** The mouse GBM neurosphere culture (ID: Ms7080) was seeded in methylcellulose-containing semisolid media and incubated under doxycycline for five days. After ionizing radiation, the GBM cells were further incubated for five days and number of neurospheres in each well (as representative images shown in top) was counted (bottom).

Increased expression of antioxidant proteins has been hypothesized to be responsible for radio-resistance of GBMs [29]. Therefore, we next tested whether PRDX4 knockdown affects radio-resistance of GBMs by applying ionizing radiation at increasing doses to GBMs in culture and quantifying the growth of surviving cells three days after the radiation. As in Figure 4 and 5 A–C, PRDX4 knockdown itself in the absence of radiation decreased the cell survival compared to the controls. Remarkably, PRDX4 knockdown was far more effective in killing GBM cells when they were irradiated: the extent of cell growth was further reduced 30–50% when PRDX4 knockdown was paired with radiation compared to radiation alone (Figure 5 A–C). Such an effect by PRDX4 knockdown plus radiation on GBM growth suppression was observed in all GBM neurosphere cultures tested, including two mouse (Figure 5A/B) and one human (Figure 5C) cultures. Since both radiation [30], [31] and PRDX4 knockdown can increase ROS level, we next examined whether PRDX4 knockdown additionally increased ROS level in irradiated GBM cells. Indeed, Prdx4 knockdown plus 5 Gy radiation showed 0.6–1.8-fold more ROS levels than single treatments (Prdx4 knockdown alone and radiation alone, Figure 5D), suggesting that additionally increased ROS levels may underlie enhanced growth suppression by the combined treatment compared to single treatments. Prdx4 knockdown plus radiation also significantly suppressed colony formation compared to single treatments in a clonogenic assay (Figure 5E), in which neurosphere formation from individual single cells was measured in methylcellulose-containing semisolid media [19]. These results suggest that reducing PRDX4 expression can potentially enhance radiation sensitivity of the GBMs.

### Reducing Prdx4 expression significantly prolonged mouse survival in an orthotopic transplantation model

**Figure 6:**
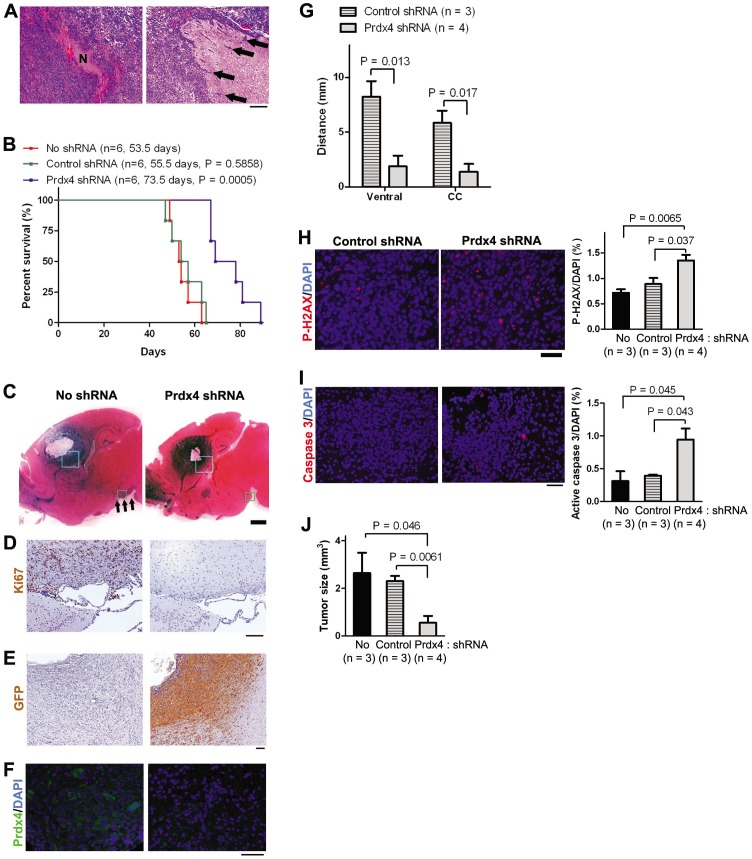
Prdx4 knock-down by shRNA induction increases mouse survival in an orthotopic model. **A,** Representative H/E images show a necrosis (N, left) in GBM and infiltration of tumor cells (arrows) into a normal brain area in symptomatic wt mice intracranially injected with 10^5^ of Mut6 GBM neurosphere-forming cells. Scale bar = 200 µm. **B,** Kaplan-Meier curves display survivals of wt mice after the intracranial injection of Mut6 GBM cells (ID: Ms6989) and doxycycline treatment. Labels indicate mouse number, mean survival, and statistic significance versus No shRNA group. **C,** Representative H/E images show brain sections from wt mice four weeks after the intracranial injection of Mut6 GBM cells. Arrows point to tumor cell infiltration into the ventral brain region. Scale bar = 1 mm. **D,** Representative IHC images show Ki67 immunoreactivity in wt mouse brains four weeks after the GBM cell injection. The brain sections were adjacent to the green boxes in panel **C**. Scale bar = 100 µm. **E,** Representative IHC images show GFP immunoreactivity in wt mouse brains four weeks after the GBM cell injection. The brain sections were adjacent to the blue boxes in panel **C**. Scale bars = 100 µm. **F,** Representative IHC images display Prdx4 immunoreactivity in wt mouse brains four weeks after the GBM cell injection. Scale bars = 50 µm. **G,** Distances of GFP immunoreactivity from the edge of tumor cell injection to the corpus callosum (CC) or ventral brain area in wt mouse brains four weeks after the GBM cell injection. **H–I,** Left: Representative IHC images show P-H2AX (**H**) or active caspase 3 (**I**) immunoreactivity in wt mouse brains four weeks after the GBM cell injection. Scale bar = 50 µm. Right: cell counting in the tumor sections. **J,** Tumor sizes in wt mouse brains four weeks after the GBM cell injection.

We next asked whether knocking down PRDX4 among GBMs *in vivo* will elicit similar effects as we had observed *in vitro* studies. To accurately assess the effect of Prdx4 knockdown in GBM phenotypes, we employed a syngenic orthotopic model, in which neurosphere-forming GBM cells from Mut6 mouse were injected into the striatum of normal mice. Such a strategy recapitulated GBM phenotypes, including necrosis and tumor cell infiltration into neighboring parenchyma (Figure 6A). Prior to injection, Mut6 GBM cells were transduced with the same lentivirus as we used in Figures 3–5. Expression of Prdx4 shRNA was induced by delivering doxycycline through drinking water from the third day of the tumor cell injection. Prdx4 knockdown in the transplanted GBM cells increased survival of recipient mice by 35% compared to control groups (Figure 6B): While the mice in control groups survived on average 55 days after the tumor cell injection, mean survival of the mice with Prdx4 knockdown was approximately 74 days. To test whether Prdx4 knockdown affected the growth and/or infiltration of the injected cells, we analyzed asymptomatic mice four weeks after the orthotopic tumor cell injection (n = 3 per treatment group). At this stage, all brain tumors in control groups showed spreading from the injection site to the ventral part of the brain (arrows in Figure 6C). These infiltrating cells were strongly Ki67-positive, suggesting that they were actively proliferating (Figure 6D). In contrast, the brain sections from the mice with Prdx4 knockdown showed substantially decreased tumor cell spreading and Ki67 staining was not detectable in the ventral regions. The presence of GFP immunoreactivity in the tumor cells in Prdx4 shRNA group but not in No shRNA group suggests induction of shRNA expression (Figure 6E). A majority of GFP immunoreactivity co-localized with a cell proliferation marker proliferating cell nuclear antigen (PCNA) in control shRNA group (Figure S6), suggesting shRNA expression in proliferating GBM cells. Successful knockdown of Prdx4 was also demonstrated by a decrease in Prdx4 immunoreactivity among tumor cells in Prdx4 shRNA groups compared to those in control groups (Figure 6F). To further characterize GBM cell spreading, we analyzed serial parasagittal sections and measured the maximum distance where GFP immunoreactivity was detected away from the tumor cell injection site. Prdx4 knockdown significantly decreased GBM cell spreading to both the ventral area and corpus callosum (Figure 6G), suggesting an inhibitory effect on GBM cell migration. However, Prdx4 knockdown did not significantly change GBM cell motility when tested in an *in vitro* cell dispersion assay on nanofiber scaffolds (Figure S7). On the other hand, immunoreactivity for P-H2AX and active (cleaved) caspase 3, markers for DNA damage and apoptosis, respectively, was higher in tumor sections with Prdx4 knockdown than in those from control groups (Figure 6H/I). These data suggest that increased DNA damage and cell death in GBM cells by Prdx4 knockdown as seen from *in vitro* data (Figure 3) may underlie the decreased tumor cell spreading *in vivo*. In support of this, the average size of transplanted tumors was smaller in Prdx4 shRNA group than those in the control groups four weeks after the orthotopic injection (Figure 6J). These results suggest that decreased GBM cell growth and infiltration by Prdx4 knockdown underlies the increased mouse survival in Prdx4 shRNA group. Taken together, our data from *in vivo* Prdx4 knockdown experiments indicate that Prdx4 overexpression contributes to GBM-related death in a mouse model.

## Discussion

We previously reported mouse genetic and neurosphere models of GBM with somatic mutations frequently found in human gliomas [11]. We have also demonstrated that NSC population is an origin cell type of the brain tumor [19]. In extension of the previous studies, we sought to identify a tumor driver, physiologically relevant to a majority of GBMs. By employing genomic screening on the mouse model and TCGA human data, we find that PRDX4 is overexpressed in a majority of GBMs. By employing shRNA-mediated gene knockdown experiments on multiple GBM cultures and an *in vivo* orthotopic transplantation model, we have shown that reducing PRDX4 expression limited growth and radio-resistance of GBM cells. Therefore, our data indicate that PRDX4 overexpression contributes to the malignant properties of GBMs.

### ROS and potential role of PRDX4 overexpression in oncogenesis

PRDX4 is composed of 271 amino acids and contains two peroxide catalysis cysteine motifs conserved in PRDX families [12]. Previous reports suggest that PRDX4 can contribute to cell survival by anti-oxidant activity against ROS. Histone H3 demethylase Ndy1 protects mouse embryonic fibroblasts (MEFs) from H_2_O_2_-mediated apoptosis with an increase in Prdx4 level [24], while TNF-related apoptosis-inducing ligand (TRAIL) decreases PRDX4 level [32]. In addition, a recent report showed that PRDX4 participates in oxidative protein folding in the endoplasmic reticulum [33]. PRDX4 is expressed in diverse cell lines and tissues, including the brain. Although high levels of PRDX4 expression have been detected in multiple human cancers [15]–[18], the role of this anti-oxidant protein in oncogenesis has just begun to be explored. A recent report demonstrated that an anti-oxidant protein sulfiredoxin (SRX) is highly expressed in human lung cancers and knockdown of SRX reduces anchorage-independent colony formation, cell migration, and invasion of human lung cancer cells [34]. SRX also interacts with PRDX4 preferentially and knockdown of PRDX4 results in similar effects, suggesting a tumor driver role in lung cancers. However, the role of PRDX4 overexpression in radio-resistance of cancer cells or in GBM phenotypes has not been reported.

Accumulating reports indicate that ROS function as important physiological regulators of diverse intracellular signaling pathways, such as growth factor stimulation and inflammatory responses [35]. Dysregulated ROS signaling has been associated with many human diseases, including cancers. Excessive accumulation of ROS (oxidative stress) is often found in human cancers and has been known to induce carcinogenesis through both genetic and epigenetic mechanisms [36]. Intriguingly, tumor suppressor mutations or oncogene activation increase intracellular ROS levels in normal or transformed, non-tumorigenic cells [21]–[23]. Therefore, it is likely that a tumor initiating mutation, such as *TP53* mutation, increases intracellular ROS level in a tumor origin cell, which in turn promotes genetic instability and further mutations. In support of this, increased ROS levels are present at precancerous stages along with *TP53* mutation [23]. It has been reported that ROS-induced oxidative stress upregulates PRDX expressions [24]. Since oxidative stress can cause cellular senescence and apoptosis, increased expression of the anti-oxidant proteins appears to be a protecting mechanism for the tumor origin or tumor cells with increased ROS. If this is true, targeting a predominant anti-oxidant protein could potentially render tumor cells to self-destruct.

### GBM driving role of PRDX4 overexpression

Many GBM cell lines survive well in spite of high levels of oxidative stress [37]. We find that PRDX4 knockdown decreased growth of multiple GBM neurosphere cultures with increases in ROS, DNA damage, and apoptosis. Therefore, our data suggest that PRDX4 overexpression protects GBM cells from oxidative stress-mediated apoptosis by regulating ROS level. This is in line with a previous study using lung cancer models [34], suggesting that PRDX4 overexpression contributes to multiple cancer types. Our data additionally suggest that PRDX4 overexpression contributes to DNA damage control and radio-resistance of GBM cells. Although many GBMs are resistant to radiation, the underlying radio-resistance mechanism has not been fully characterized. Increased expression of antioxidant proteins after radiation [29], [38] suggests that overexpression of antioxidant proteins may confer radio-resistance to GBM cells. In support of this, previous studies have shown that antioxidant proteins catalase and PRDX2 are also highly expressed in glioma cells [26], [39]. Inhibition of catalase activity and decreasing PRDX2 expression sensitized glioma cells to oxidative stress and ionizing radiation, respectively. In addition to these findings, our study demonstrates that PRDX4 knockdown significantly enhanced radiation-mediated suppression of GBM cell growth, strengthening the hypothesis that overexpression of antioxidant proteins confers radio-resistance to GBM cells.

### Targeting PRDX4 overexpression or the stress response to ROS as a future GBM therapy

PRDX4 knockdown did not significantly reduce growth of MEFs [33] and our data indicate that PRDX4 expression was increased highly in human GBMs compared to normal brain tissues. These may suggest that PRDX4 overexpression can be an attractive target in future GBM therapies. Our study showed that PRDX4 knockdown increased intracellular ROS, DNA damage, and apoptosis in GBM cells *in vitro*. The effects of PRDX4 knockdown on DNA damage and apoptosis were reproduced in an orthotopic transplantation model, resulting in suppression of GBM growth and spreading at an early stage of tumor development (four weeks after tumor cell injection) *in vivo*. Therefore, PRDX4 targeting can provide an innovative strategy for cancer treatment, utilizing a weakness of cancer cells – regulation of increased ROS level. Especially, the decreased GBM cell growth when radiation was combined with PRDX4 knockdown suggests that PRDX4 targeting would enhance the effect of radiation therapy on GBM patients. Because our study clearly illustrated that PRDX4 was expressed at a highly elevated level in a majority of GBMs, such PRDX4 targeting plus radiation could be utilized to treat many GBMs. In support of our concept to target PRDX4 as future GBM therapy, a small molecule (piperlongumine) which increases ROS levels in breast cancer cells induced cancer cell death *in vitro* and inhibited tumor growth *in vivo* [40]. Several other putative GBM drivers have recently been reported [41]–[46]. Further characterizations of the candidate GBM drivers would provide improved GBM therapies in the future.

In summary, we have shown that PRDX4 overexpression contributes to growth and radio-resistance of multiple GBM cultures. Because we also found that PRDX4 was expressed at a substantially increased level in a majority of human GBMs, our data suggest that PRDX4 overexpression may drive malignant properties of many GBMs. We also presented that suppression of PRDX4 expression reduced growth and radio-resistance GBMs, providing PRDX4 as a novel target for future GBM therapies.

## Materials and Methods

### Mice

Animal study was carried out in strict accordance with the recommendations in the Guide for the Care and Use of Laboratory Animals of the National Institutes of Health. All animal experiments were approved by the Committee on the Institutional Animal Care and Use Committee at the Ohio State University (Permit Number: 2008A0192-R1). We purchased B6CBAF1/J female mice (wild-type, wt) from Jackson Laboratory and bred with Mut3 (*GFAP-cre*; *Nf1* ^loxP/+^; *Trp53* ^−/+^) [11] or *Trp53* ^loxP^; *Pten* ^loxP^ male mice [47], [48] to maintain the lines. We bred Mut3 males with *Trp53* ^loxP/loxP^; *Pten* ^loxP/loxP^ female mice to produce Mut6 mice as described [11].

### Neurosphere culture

We established and maintained neurosphere cultures from mouse GBMs and the SVZ as described [11], except using EGF from Daewoong Pharmaceutical Co., Ltd (Yongin, Gyeonggi-do, Korea) and TrypLE (Invitrogen). We used human patient-derived GBM neurospheres previously established and maintained under the protocols approved by the Ohio State University Institutional Review Board (OSU IRB) [49].

### Quantitative real time PCR (qRT-PCR)

We isolated total RNA using RNeasy Lipid Tissue Mini Kit (Qiagen). To generate cDNA, we reverse transcribed 1 µg of total RNA using High Capacity cDNA Reverse Transcription Kit (Applied Biosystems). After serially diluting cDNA with nuclease free water, we mixed the samples with iQ™ SYBR Green Supermix (Bio-Rad Laboratories) and primers (Table S2). We performed PCR amplifications and quantified data using iQ5 Multicolor RT-PCR Detection System (Bio-Rad). We obtained relative quantification using ΔΔCt method with GAPDH as an endogenous control and presented data as relative gene expression, a ratio of target gene expression to reference control.

### Lentivirus-mediated, inducible shRNA expression

Generation of lentivirus expressing shRNA under the Tet-U6 promoter is described in Supplementary Materials and Methods S1. To knock-down PRDX4, we seeded 5.0×10^4^ of neurosphere-forming GBM cells per well in 6-well plates and added lentivirus-containing media supplemented with 8.0 µg/ml polybrene (Sigma) next day. After one day of incubation, we washed the cells and replenished with fresh media containing 5 µg/ml doxycycline (Sigma) to express shRNA. After three days of incubation, we sorted EGFP-expressing cells by using a FACSAria flow cytometer (Becton-Dickson). We used the sorted cells for further assays by maintaining them at 5 µg/ml of doxycycline.

### Cell growth and ROS assays

We plated 5.0×10^3^ of neurosphere-forming cells per well in 96-well plates and incubated two to six days. After taking representative images using an inverted microscope (Olympus IX50, Olympus America), we assessed cell growth by using CellTiter 96 Aqueous One Solution Cell Proliferation Assay kit (Promega) and measuring optical density at 490 nm in Multiskan Spectrum 96-well plate reader (Thermo Fisher Scientific). This assay utilizes reduction of a tetrazolium compound MTS by dehydrogenase enzymes in metabolically active cells and resulted in similar data to direct cell counting by Vi-cell viability analyzer (Beckman Coulter, Brea, CA) (Figure S8). To assess the effect of antioxidant NAC on GBM cell growth, we plated 1.0×10^5^ of neurosphere-forming cells per well in 24-well plates and treated with 0–10 mM of NAC (Calbiochem, Darmstadt, Germany) next day. We incubated the cells for three days in the presence of doxycycline (5 µg/ml) and measured cell growth by direct cell counting using Vi-cell viability analyzer.

To measure cellular ROS levels, we employed an ROS-sensitive dye DCFDA that has been frequently used to assess activity of PRDX family members [50]–[54]. We seeded 1.0×10^5^ of neurosphere-forming cells per well in 6-well plates and incubated for three days for mouse GBM cells. After harvesting the cells, we dissociated them into single cell suspension with TrypLE and incubated with 10 µM of ROS-sensitive dye chloromethyl-H_2_ DCFDA (Invitrogen) for 20 min in the dark at 37°C. As negative control, subsets of cells were incubated with 1 µM of ROS-insensitive probe carboxy-DCFDA (Invitrogen) instead of chloromethyl-H_2_ DCFDA for 10 min as described [26]. After washing and resuspending the cells in PBS, we assessed oxidation of the dye at 520 nm by measuring 10,000 events per sample using a FACSCalibur flow cytometer (Becton-Dickinson).

### Comet assay and radiation

We assessed DNA damage by using comet assay (alkaline single cell gel electrophoresis assay) according to the manufacturer's instruction (Trevigen Inc, Gaithersburg, MD). Briefly, we mixed 1.0×10^6^/ml of GBM cells in PBS with low melting agarose at a ratio of 1∶10 (v/v) and immediately plated 50 µl of the mixture onto Cometslide™ to immobilize the cells. After treating with lysis and alkaline unwinding solutions, we placed the slides in electrophoresis tray and performed electrophoresis at 21 V for 30 min. After washing with distilled water and 70% ethanol, we dried the samples and added 100 µl of SYBR green I solution. We prepared three slides for each sample and evaluated at least 100 cells per sample by using epifluorescence microscopy (Nikon Eclipse TE2000, Melville, NY). We measured comet length by using MetaMorph software (Universal Imaging Corporation).

For radiation assay, we plated 5×10^3^ of GBM cells per well in 96-well plates and incubated for three days. Then, we irradiated the cells by using RS 2000 Biological irradiator (Rad Source Technologies) delivering a mean dose rate of 110 cGy/min. GBM cells were exposed to an individual total dose of 2, 5, or 10 Gy, corresponding to irradiation time of 109, 272, or 545 s, respectively. We assessed cell growth three days after further incubation. To measure ROS levels after radiation, we plated 2.0×10^5^ cells in 25 cm^2^ flasks (Nunc, Rochester, NY) and incubated in a doxycycline-containing media (5 µg/ml). We then irradiated the cells as described above and incubated for three days. We assessed ROS levels by incubating the cells with chloromethyl-H_2_ DCFDA or negative control carboxy-DCFDA and performing FACS analysis as described above. To assess clonogenic survival of GBM cells after radiation, we combined irradiation with a self-renewal assay for stem cells, which measures neurosphere formation from individual cells in a semisolid media [19]. After plating 5.0×10^2^ cells per well in 96-well plates in a media containing 0.8% methylcellulose (Sigma), we incubated the cells for five days. We then irradiated the cells as described above and incubated for five more days. We took pictures of each well using an inverted microscope (Nikon Eclipse Ti, Melville, NY) and counted neurosphere numbers in a blind way by using MetaMorph software.

Additional Materials and Methods are described in Supplementary Information Materials and Methods S1.

## Supporting Information

Figure S1 Genomic analyses reveal functions of the genes dysregulated in both mouse and human GBMs. IPA on the genes differentially expressed in both mouse and human GBMs categorized the genes with similar functions. Six gene functions with highest statistic significance are shown.
(PDF)

Figure S2 Prdx4 is highly expressed in Mut6 mouse GBMs. Representative three GBM images from four Mut6 mice are shown in comparison with matched normal brain regions in wt mice. By cell counting on randomly chosen fields, Prdx4 immunoreactivity was detected in 30.16±6.42% (mean ± s.e.m., n = 4) of cells in Mut6 GBM areas. Scale bar = 50 µm.
(PDF)

Figure S3 Prdx4 knock-down by shRNA increases ROS level in mouse GBM neurospheres. Left: FACS analysis data after incubation with ROS-sensitive chloromethyl-H_2_ DCFDA (CM-DCFDA) compare ROS levels in a Mut6 mouse GBM neurosphere culture (ID: Ms7080) three days after seeding and doxycycline treatment. Right: FACS analysis data after incubating the same mouse GBM neurosphere culture with ROS-insensitive carboxyl-DCFDA (C-DCFDA) as a negative control of the assay. Note that these data were from an independent experiment for Figure 3D.
(PDF)

Figure S4 Two different Prdx4 shRNAs significantly suppress Prdx4 expression and tumor cell growth in GBM neurospheres. **A,** Representative qRT-PCR analysis displays relative Prdx4 expression three days after doxycycline treatment to induce shRNA expression in a Mut6 mouse GBM neurosphere culture (ID: Ms6989). **B,** Representative MTS assay data show growth of the mouse GBM neurosphere culture three days after the shRNA-mediated Prdx4 knockdown.
(PDF)

Figure S5 Prdx4 knockdown does not significantly change cell cycle of mouse GBM neurosphere cultures. Flow cytometry analysis displays relative numbers of mouse GBM cells (ID: Ms6989) at each cell cycle phase three days after doxycycline-induced shRNA expression. n.s.: no significant change (P>0.4).
(PDF)

Figure S6 Expression of shRNA is induced among proliferating GBM cells in a transplantation model. Representative IHC images display immunoreactivity for GFP and PCNA in wt mouse brains four weeks after intracranial injection of GBM cells (ID: Ms6989) expressing control shRNA. Scale bar = 50 µm.
(PDF)

Figure S7 Prdx4 knockdown does not significantly change GBM cell migration on nanofiber scaffolds. A mouse GBM neurosphere line (ID: Ms7080) expressing shRNA was analyzed in a cell migration assay using aligned-nanofiber scaffolds. Left: Representative GBM neurosphere cells visualized under fluorescence field at 0 or 24 hours after plating on nanofiber-coated plates. Scale bar = 500 um. Right: maximal linear dispersion (Fmax) of the GBM cells 24 hours after plating, expressed as a ratio to the original diameter of the neurospheres.
(PDF)

Figure S8 MTS assay and direct cell counting show similar results in assessing GBM cell growth. Growth of mouse GBM neurospheres (ID: Ms7080) was measured by two different assays three days after shRNA-mediated Prdx4 knockdown. Representative assay data show relative growth of the neurospheres, measured by either CellTiter 96 Aqueous One Solution Cell Proliferation Assay (left, MTS assay) or direct cell counting using Vi-cell viability analyzer (right: cell counting).
(PDF)

Table S1 Expression fold-changes of 22 genes in the top-ranked network by IPA on human and mouse GBM gene expression analysis.
(PDF)

Table S2 Sequences of qRT-PCR primers and shRNAs.
(PDF)

Materials and Methods S1 Supporting materials and methods.
(PDF)
