# Supplementary figures and images for: Suppression of Peroxiredoxin 4 in Glioblastoma Cells Increases Apoptosis and Reduces Tumor Growth
Source: PLoS One. 2012 Aug 15;7(8):e42818. doi: 10.1371/journal.pone.0042818 (PMC3419743; doi:10.1371/journal.pone.0042818)

# Kim et al., Supplementary Figure S1

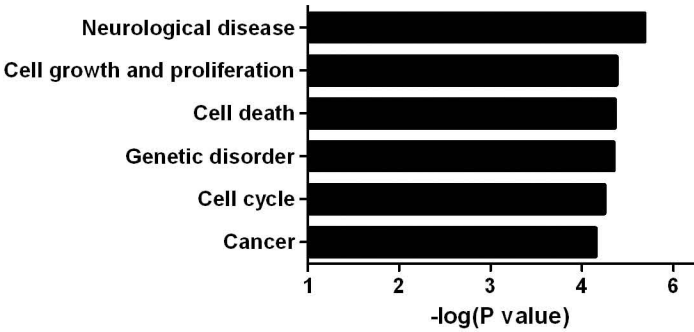

Supplement: Figure S1 — Genomic analyses reveal functions of the genes dysregulated in both mouse and human GBMs. IPA on the genes differentially expressed in both mouse and human GBMs categorized the genes with similar functions. Six gene functions with highest statistic significance are shown. (PDF) [file pone.0042818.s001.pdf]

# Kim et al., Supplementary Figure S2

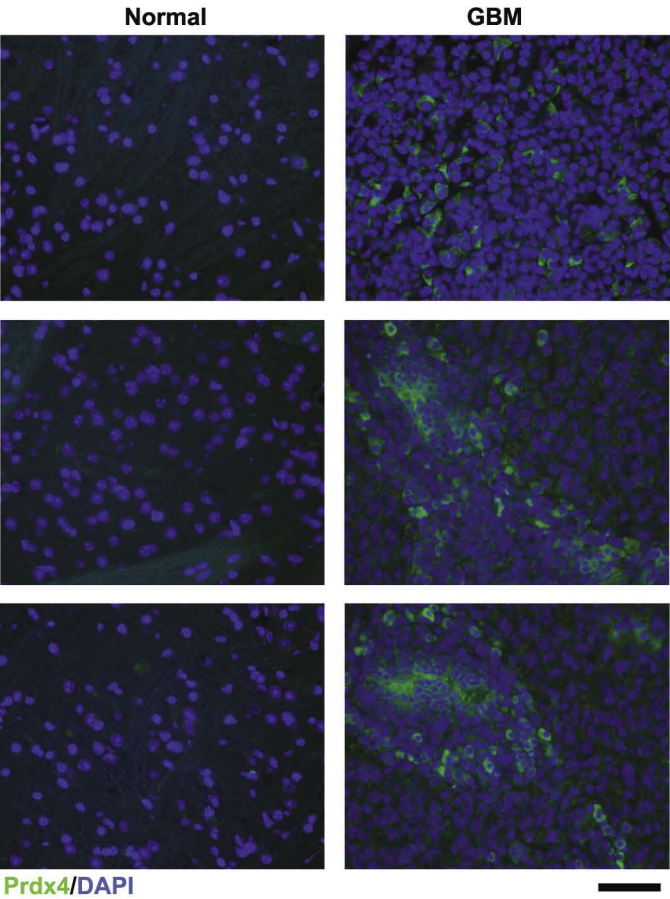

Supplement: Figure S2 — Prdx4 is highly expressed in Mut6 mouse GBMs. Representative three GBM images from four Mut6 mice are shown in comparison with matched normal brain regions in wt mice. By cell counting on randomly chosen fields, Prdx4 immunoreactivity was detected in 30.16±6.42% (mean ± s.e.m., n = 4) of cells in Mut6 GBM areas. Scale bar = 50 µm. (PDF) [file pone.0042818.s002.pdf]

# Kim et al., Supplementary Figure S3

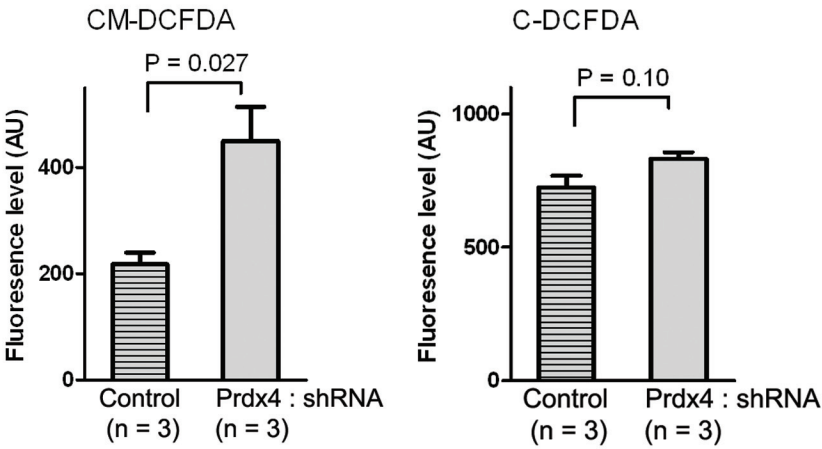

Supplement: Figure S3 — Prdx4 knock-down by shRNA increases ROS level in mouse GBM neurospheres. Left: FACS analysis data after incubation with ROS-sensitive chloromethyl-H2 DCFDA (CM-DCFDA) compare ROS levels in a Mut6 mouse GBM neurosphere culture (ID: Ms7080) three days after seeding and doxycycline treatment. Right: FACS analysis data after incubating the same mouse GBM neurosphere culture with ROS-insensitive carboxyl-DCFDA (C-DCFDA) as a negative control of the assay. Note that these data were from an independent experiment for Figure 3D. (PDF) [file pone.0042818.s003.pdf]

# Kim et al., Supplementary Figure S4

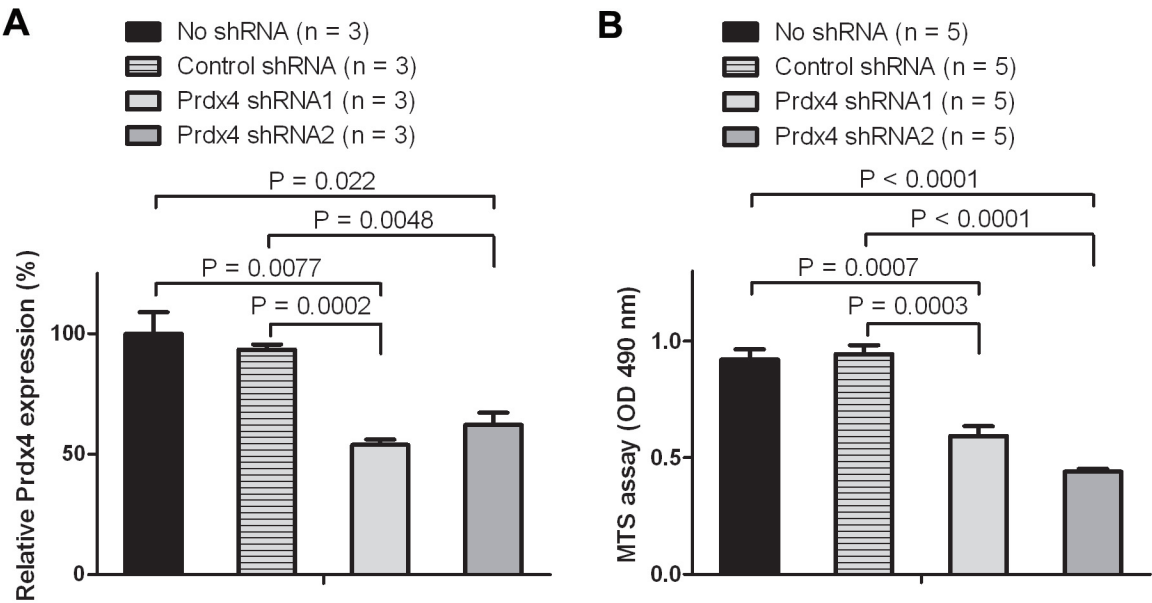

Supplement: Figure S4 — Two different Prdx4 shRNAs significantly suppress Prdx4 expression and tumor cell growth in GBM neurospheres. A, Representative qRT-PCR analysis displays relative Prdx4 expression three days after doxycycline treatment to induce shRNA expression in a Mut6 mouse GBM neurosphere culture (ID: Ms6989). B, Representative MTS assay data show growth of the mouse GBM neurosphere culture three days after the shRNA-mediated Prdx4 knockdown. (PDF) [file pone.0042818.s004.pdf]

# Kim et al., Supplementary Figure S5

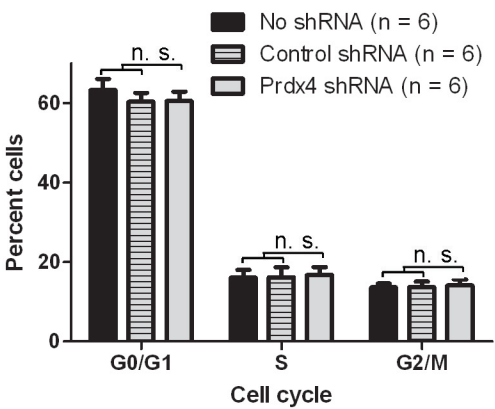

Supplement: Figure S5 — Prdx4 knockdown does not significantly change cell cycle of mouse GBM neurosphere cultures. Flow cytometry analysis displays relative numbers of mouse GBM cells (ID: Ms6989) at each cell cycle phase three days after doxycycline-induced shRNA expression. n.s.: no significant change (P>0.4). (PDF) [file pone.0042818.s005.pdf]

# Kim et al., Supplementary Figure S6

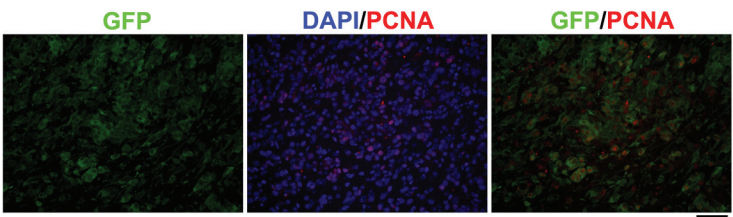

Supplement: Figure S6 — Expression of shRNA is induced among proliferating GBM cells in a transplantation model. Representative IHC images display immunoreactivity for GFP and PCNA in wt mouse brains four weeks after intracranial injection of GBM cells (ID: Ms6989) expressing control shRNA. Scale bar = 50 µm. (PDF) [file pone.0042818.s006.pdf]

# Kim et al., Supplementary Figure S7

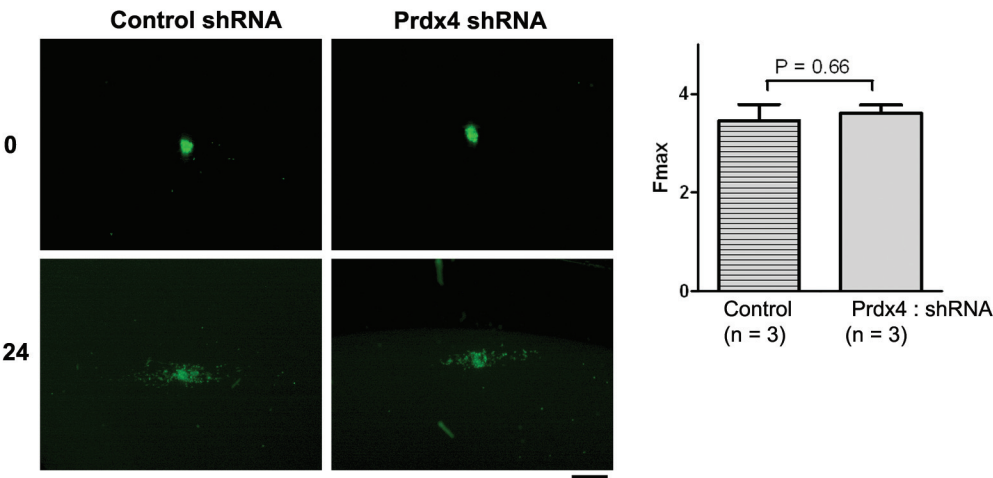

Supplement: Figure S7 — Prdx4 knockdown does not significantly change GBM cell migration on nanofiber scaffolds. A mouse GBM neurosphere line (ID: Ms7080) expressing shRNA was analyzed in a cell migration assay using aligned-nanofiber scaffolds. Left: Representative GBM neurosphere cells visualized under fluorescence field at 0 or 24 hours after plating on nanofiber-coated plates. Scale bar = 500 um. Right: maximal linear dispersion (Fmax) of the GBM cells 24 hours after plating, expressed as a ratio to the original diameter of the neurospheres. (PDF) [file pone.0042818.s007.pdf]

# Kim et al., Supplementary Figure S8

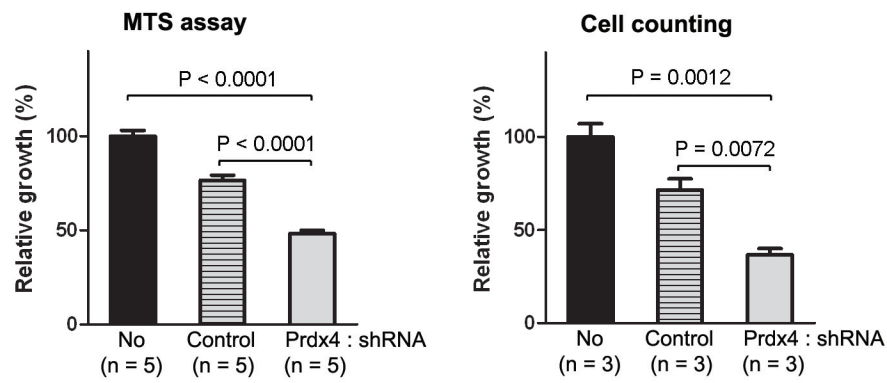

Supplement: Figure S8 — MTS assay and direct cell counting show similar results in assessing GBM cell growth. Growth of mouse GBM neurospheres (ID: Ms7080) was measured by two different assays three days after shRNA-mediated Prdx4 knockdown. Representative assay data show relative growth of the neurospheres, measured by either CellTiter 96 Aqueous One Solution Cell Proliferation Assay (left, MTS assay) or direct cell counting using Vi-cell viability analyzer (right: cell counting). (PDF) [file pone.0042818.s008.pdf]
