# Supplementary material for: Suppression of Peroxiredoxin 4 in Glioblastoma Cells Increases Apoptosis and Reduces Tumor Growth
Source: PLoS One. 2012 Aug 15;7(8):e42818. doi: 10.1371/journal.pone.0042818 (PMC3419743; doi:10.1371/journal.pone.0042818)
Supplement: Table S1 — Expression fold-changes of 22 genes in the top-ranked network by IPA on human and mouse GBM gene expression analysis. (PDF) [file pone.0042818.s009.pdf]

Supplementary Table S1. Expression fold changes of 22 genes in the top-ranked network by IPA on human and mouse GBM gene expression analysis.

Gene Symbol	Human data		Mouse data	
	Fold change (GBM/normal)	P value	Fold change (GBM/NSC)	P value
G3BP2	-1.793	0.0000001	-2.533	0.0000576
TXNIP	3.187	< 1e-07	2.529	0.0000754
PRDX4	5.037	< 1e-07	1.600	0.0000993
BTRC	-1.860	< 1e-07	-1.846	0.0014054
RPS6KA5	-3.055	< 1e-07	-1.882	0.0014759
TFPI	19.832	< 1e-07	9.603	0.0018126
GLI2	1.591	0.0007228	2.684	0.0018847
NFKBIA	1.813	0.0001141	1.628	0.0041860
ARFGAP3	2.164	< 1e-07	1.970	0.0043959
DAXX	1.677	0.0000003	1.524	0.0046179
FSTL1	6.492	< 1e-07	2.842	0.0047270
HES1	2.622	0.0000075	1.529	0.0074982
RAP1GAP	-7.929	< 1e-07	-1.986	0.0103547
XBP1	1.859	0.0000061	1.510	0.0119999
EFNA1	2.207	0.0000001	1.845	0.0142324
VCAN	9.724	< 1e-07	1.858	0.0152890
PEA15	-1.846	0.0000004	-2.251	0.0155068
PSME1	1.940	0.0000015	1.509	0.0170793
ZC3HAV1	3.298	< 1e-07	1.629	0.0199848
FBN1	3.138	0.0005349	2.431	0.0302093
ROCK2	-1.703	0.0000025	-4.323	0.0325901
TCF4	2.019	0.0000361	1.569	0.0356192
