# Supplementary material for: Suppression of Peroxiredoxin 4 in Glioblastoma Cells Increases Apoptosis and Reduces Tumor Growth
Source: PLoS One. 2012 Aug 15;7(8):e42818. doi: 10.1371/journal.pone.0042818 (PMC3419743; doi:10.1371/journal.pone.0042818)
Supplement: Table S2 — Sequences of qRT-PCR primers and shRNAs. (PDF) [file pone.0042818.s010.pdf]

Supplementary Table S2. Sequences of qRT-PCR primers and shRNAs.

Purpose/species	Name	Sequence
qRT-PCR/mouse	Gapdh F	5′-CGTCCCGTAGACAAAATGGT-3′
”	Gapdh R	5′-TTGATGGCAACAATCTCCAC-3′
”	Prdx4 F	5′-CGGATCACTCCCTGCATCTA-3′
”	Prdx4 R	5′-TGAGCTCCTTGAATTCTCCG-3′
qRT-PCR/human	GAPDH F	5′-AAGGTGAAGGTCGGAGTCAA-3′
”	GAPDH R	5′-AATGAAGGGGTCATTGATGG-3′
”	PRDX4 F	5′-TTGGCGACAGACTTGAAGAA-3′
”	PRDX4 R	5′-ATCCTTATTGGCCCAAGTCC-3′
shRNA/mouse	Prdx4 shRNA 1	5′-GGAAGGAACAGCTGTGATTAA-3′
”	Prdx4 shRNA 2	5′-GAAGTATTTCGACAAGCTAAA-3′
shRNA/human	PRDX4 shRNA	5′-GAAACCTGGTAGTGAAACAAT-3′
