# Supplementary material for: Suppression of Peroxiredoxin 4 in Glioblastoma Cells Increases Apoptosis and Reduces Tumor Growth
Source: PLoS One. 2012 Aug 15;7(8):e42818. doi: 10.1371/journal.pone.0042818 (PMC3419743; doi:10.1371/journal.pone.0042818)
Supplement: Materials and Methods S1 — Supporting materials and methods. (PDF) [file pone.0042818.s011.pdf]

Supplementary Information

Supplementary Materials and Methods

Microarray and data analysis

We extracted total RNA from wild type (wt) subventricular zone (SVZ) and Mut6 glioma neurospheres (n = 3 per group) using Trizol (Invitrogen) for the global gene expression profiling performed in the core facility at The University of Texas Southwestern Medical Center (Illumina Mouse-6-v1 expression arrays, Illumina Inc). We performed gene expression data analysis using Illumina Bead Studio software version 1.5.0.34. We used the cubic-spline method to normalize intensities to remove any non-biological variation between samples. We considered a gene expression detected if the detection P value was lower than 0.05. We performed differential expression analysis comparing wt SVZ and Mut6 glioma groups by taking wt SVZ group as the reference in Illumina Custom error model and calculated Diff-scores based on bead standard deviation. We considered a gene expression significantly altered in Mut6 if the |Diff-score| > 13 (equivalent to P < 0.05). We obtained 1170 genes from the data mining. The microarray data have been submitted to the Gene Expression Omnibus repository (GEO) at www.ncbi.nlm.nih.gov/geo/ and assigned the accession number GSE34333.

From the TCGA database (http://tcga-data.nci.nih.gov/tcga/), we obtained 272 human GBM and 10 normal samples (from Batches 1 – 8 on April 2009) processed with Affymetrix HG-U133A arrays. We obtained gene expression estimates using Robust

Multi-array Average method after quantile normalization. To identify genes that are differentially expressed between GBM and normal groups, we applied t-test based on random variance mode using BRB-Array Tool software [1]. We consider a gene with P value < 0.001, fold difference over 1.5, and controlling FDR at 0.0025 to be significantly altered between two groups. We obtained 4790 genes from the data mining. We mapped the differentially expressed gene list obtained from Illumina mouse expression analysis to genes in Affymetrix human arrays using mouse-human homology maps from NCBI (http://www.ncbi.nlm.nih.gov/projects/homology/maps/). We found that 147 genes were commonly altered in both mouse and human GBMs. We used IPA software (www.ingenuity.com) for the network and functional analysis on the differentially expressed genes.

Histology

We purchased human GBM tissue arrays (GL804 and GL806a) from US Biomax, Inc and additionally obtained fresh human GBM samples from patients under the protocols approved by the OSU IRB. We dissected out mouse brains, processed both human and mouse brain specimens for paraffin embedding, and cut into 5-μm sections as previously described [2]. We performed all immunohistochemistry (IHC) on triplicate or more brain sections per group. In case of mouse brain sections, we chose matched sections from controls and samples, based on anatomy of the hippocampus and SVZ. Antibodies used for IHC were against PRDX4 (AF5460, R and D Systems), Ki67 antigen (VP-K451, Vector Laboratories), GFP (GFP-1020, Aves lab), PCNA (MAB4078, Millipore), P-H2AX (2577, Cell Signaling), or active caspase 3 (C92-605, BD Pharmingen). We used

microwave antigen retrieval for antibodies against PRDX4 and Ki67 antigen. We amplified and visualized the primary antibodies as described [3].

To assess tumor cell spreading *in vivo*, we took 2X pictures of brain sections stained for GFP immunoreactivity and blindly measured the longest distances of GFP immunoreactivity from the edge of tumor cell injection to the corpus callosum or ventral brain area by using MetaMorph software (Universal Imaging Corporation). We blindly counted active caspase 3/DAPI-positive cells in three or four randomly chosen 0.44 X 0.33 mm^2 fields within tumor-containing brain sections by using MetaMorph software. To measure tumor size, we first took pictures of H/E-stained every fifth section of mouse brains injected with GBM cells by using an inverted microscope (Nikon ECLIPSE Ti, Melville, NY). We then blindly measured tumor volume by using ImageJ (http://rsbweb.nih.gov/ij/) with Volumest plug-in.

Western blotting analysis

All studies involving human specimens were performed in compliance with the guidelines of the Human Investigations Committee at The Ohio State University School of Medicine. Pathologically-graded GBM specimens (patient age ranging from 32 to 70 years) were obtained after surgery and immediately frozen in liquid nitrogen. Normal tissue samples were derived from non-diseased tissue obtained during epilepsy surgery or from donor tissue with post-mortem interval shorter than 12 hrs. Frozen samples were thawed in a lysis buffer: 20 mM Tris-HCl, pH 8.0, containing 137 mM NaCl, 1% w/v NP-40, 1 mM $MgCl_2$, 10% w/v glycerol, 0.5 mM EDTA, 10 mM $Na_2P_2O_7$, 10 mM NaF, 1 μg/ml aprotinin, 10 μg/ml leupeptin, 1 mM vanadate, and 1 mM phenylmethylsulfonyl

fluoride, and subsequently homogenized using Dounce homogenizer. We prepared lysates of neurospheres and performed Western blotting as described [2]. Antibodies: PRDX4 (AF5460) was from R & D Systems, β-actin (AC-15) from Sigma, and P-H2AX (9718) and H2AX (2595) from Cell Signaling. We analyzed band densities using Molecular Dynamics Personal Densitomer SI and Image Quant software (Amersham Biosciences).

Lentivirus

To induce gene knockdown, we used the tetracycline-dependent lentiviral vector pLVET-tTR-KRAB [4]. This plasmid carries a hybrid Tet-U6 promoter to regulate expression of shRNA as well as the chimeric repressor TetR-KRAB that shuts down gene expression and is inhibited by tetracycline or doxycycline. To make the lentiviral vector, we annealed oligonucleotide sequences specific for PRDX4 shRNAs (Table S2) or the universal negative control sequence AllStars shRNA (Qiagen) and cloned between Mlu I and Cla I sites in the shuttle vector pLVTHM. Subsequently, we excised a fragment of pLVTHM encompassing the shRNA sequence with Msc I and Xba I (New England Biolabs) and subcloned into pLVET-tTR-KRAB. To produce lentiviruses, we first seeded 5 X 10^6 of HEK293 cells in DMEM (Invitrogen) with 10% FBS in 100-mm dishes and incubated overnight. We then co-transfected 5 μg of pLVET-tTR-KRAB lentiviral vector, 2.5 μg of psPAX2 packing plasmid, and 2.5 μg of pMD2.G envelope plasmid by using FuGENE HD transfection reagent (Roche). We replaced the medium after 24 hr, harvested lentiviruses after additional 48 hr, centrifuged for 5 min at 3,000 rpm, aliquoted, and stored at -80°C. We titered lentivirus using HEK293T cells as previously described

[4] and obtained approximately 1.0 X 10^6 transducing unit viral titers per ml. We monitored shRNA expression by doxycycline-dependent co-expression of EGFP carried in the lentiviral backbone [4].

Cell cycle and death assays

To analyze cell cycle distribution after shRNA expression, we harvested GBMs cells three days after doxycycline treatment, washed with PBS, and fixed in 70% ethanol. After washing with PBS, we resuspended the cells in PBS containing 20 μg/ml propidium iodide (Sigma), 200 μg/ml RNase A, and 1% (v/v) Triton-X 100. After 30 min of incubation at room temperature, we measured cell cycle distribution using a FACSCalibur flow cytometer. To detect apoptotic cell death, we plated 1 x 10^6 of GBM cells per well in 6-well plates and incubated for three days with doxycycline (5 μg/ml). We then stained the cells with Annexin V and propidium iodide using Vybrant Apoptosis Assay Kit #6 (Molecular Probes). We analyzed the stained cells using a FACSAria flow cytometer.

Syngenic orthotopic mouse model of GBM

Animal study was carried out in strict accordance with the recommendations in the Guide for the Care and Use of Laboratory Animals of the National Institutes of Health. All animal experiments were approved by the Committee on the Institutional Animal Care and Use Committee at the Ohio State University (Permit Number: 2008A0192-R1). All surgery was performed under ketamine/xylazine anesthesia, and all efforts were made to minimize suffering. We deeply anesthetized 7 – 8-week-old female B6CBAF1/J mice

(Jackson Laboratory) by intraperitoneal injection of ketamine (100 mg/kg) and xylazine (20 mg/kg) mixture and positioned them on stereotactic injection frames. After locally shaving hairs on the head, we drilled a small hole and injected 1.0 X 10^5 of neurosphere-forming GBM cells into the striatum. The stereotactic coordination of intracranial injection was 0.5 mm anterior, 2.0 mm lateral to the bregma, and 2.75 mm intraparenchymal. To induce shRNA expression, we provided the mice with doxycycline (2 mg/ml) containing water three days after the tumor cell injection. We sacrificed the mice either four weeks after the injection or when they showed a neurological symptom of brain tumor as previously described [2].

Cell migration assay on nanofiber scaffolds

We analyzed GBM cell migration using three-dimensional nanofiber scaffolds as previously described [5]. Briefly, we stained GBM neurospheres (200 – 250 um in diameter) with 5 μM CellTacker Green CMFDA (Invitrogen) and manually placed individual neurospheres on multi-well plates containing scaffolds of highly-aligned nanofibers (Nanofiber Solutions LLC, Columbus OH). Rapid migration of cells on these scaffolds is an indicator of 3D migratory ability independently of cell proliferation or matrix invasion [5]. After incubating the cells in doxycycline-containing media for 24 hours, we measured the axial distance of cell migration. Fmax was calculated as the ratio of maximum linear dispersion divided by the original diameter of the neurosphere.

Statistical Analysis

We analyzed mouse survival by plotting Kaplan-Meier survival curves using log-rank test in GraphPad Prism S/W (GraphPad Software, Inc). For other assays we used two-sample student t-test or one-way ANOVA when more than two groups were compared. We displayed all data as mean ± s.e.m.

Supplementary References

1. Wright GW, Simon RM (2003) A random variance model for detection of differential gene expression in small microarray experiments. Bioinformatics 19: 2448-2455.
2. Kwon CH, Zhao D, Chen J, Alcantara S, Li Y, et al. (2008) Pten Haploinsufficiency Accelerates Formation of High Grade Astrocytomas Cancer Res 68: 3286-3294.
3. Kwon CH, Luikart BW, Powell CM, Zhou J, Matheny SA, et al. (2006) Pten regulates neuronal arborization and social interaction in mice. Neuron 50: 377-388.
4. Szulc J, Wiznerowicz M, Sauvain MO, Trono D, Aebischer P (2006) A versatile tool for conditional gene expression and knockdown. Nat Methods 3: 109-116.
5. Agudelo-Garcia PA, De Jesus JK, Williams SP, Nowicki MO, Chiocca EA, et al. Glioma cell migration on three-dimensional nanofiber scaffolds is regulated by substrate topography and abolished by inhibition of STAT3 signaling. Neoplasia 13: 831-840.
